# Characterization and Optimization of Chitosan-Coated Polybutylcyanoacrylate Nanoparticles for the Transfection-Guided Neural Differentiation of Mouse Induced Pluripotent Stem Cells

**DOI:** 10.3390/ijms22168741

**Published:** 2021-08-14

**Authors:** Martin Hsiu-Chu Lin, Ping-Shan Lai, Li-Ching Chang, Wei-Chao Huang, Ming-Hsueh Lee, Kuo-Tai Chen, Chiu-Yen Chung, Jen-Tsung Yang

**Affiliations:** 1 Department of Neurosurgery, Chang Gung Memorial Hospital, Chia-Yi Branch, Chia-Yi 61363, Taiwan; martinhclin@hotmail.com (M.H.-C.L.); u9201031@gmail.com (W.-C.H.); ma2072@gmail.com (M.-H.L.); tad91116@gmail.com (K.-T.C.); 2 Department of Chemistry, National Chung Hsing University, Taichung 40227, Taiwan; pslai@email.nchu.edu.tw; 3 PhD Programme of Tissue Engineering and Regenerative Medicine, National Chung Hsing University, Taichung 40227, Taiwan; 4 Department of Dentistry, Chang Gung Memorial Hospital, Chia-Yi Branch, Chia-Yi 61363, Taiwan; liching@ms39.hinet.net; 5 Department of Nursing, Chang Gung University of Science and Technology, Chia-Yi 61363, Taiwan; 6 College of Medicine, School of Traditional Chinese Medicine, Chang Gung University, Tao-Yuan 33302, Taiwan

**Keywords:** polybutylcyanoacrylate nanoparticles, chitosan, induced pluripotent stem cell, gene transfection

## Abstract

Gene transfection is a valuable tool for analyzing gene regulation and function, and providing an avenue for the genetic engineering of cells for therapeutic purposes. Though efficient, the potential concerns over viral vectors for gene transfection has led to research in non-viral alternatives. Cationic polyplexes such as those synthesized from chitosan offer distinct advantages such as enhanced polyplex stability, cellular uptake, endo-lysosomal escape, and release, but are limited by the poor solubility and viscosity of chitosan. In this study, the easily synthesized biocompatible and biodegradable polymeric polysorbate 80 polybutylcyanoacrylate nanoparticles (PS80 PBCA NP) are utilized as the backbone for surface modification with chitosan, in order to address the synthetic issues faced when using chitosan alone as a carrier. Plasmid DNA (*p*DNA) containing the brain-derived neurotrophic factor (BDNF) gene coupled to a hypoxia-responsive element and the cytomegalovirus promotor gene was selected as the genetic cargo for the in vitro transfection-guided neural-lineage specification of mouse induced pluripotent stem cells (iPSCs), which were assessed by immunofluorescence staining. The chitosan-coated PS80 PBCA NP/BDNF *p*DNA polyplex measured 163.8 ± 1.8 nm and zeta potential measured −34.8 ± 1.8 mV with 0.01% (*w*/*v*) high molecular weight chitosan (HMWC); the *p*DNA loading efficiency reached 90% at a nanoparticle to *p*DNA weight ratio of 15, which also corresponded to enhanced polyplex stability on the DNA stability assay. The HMWC-PS80 PBCA NP/BDNF *p*DNA polyplex was non-toxic to mouse iPSCs for up to 80 μg/mL (weight ratio = 40) and enhanced the expression of BDNF when compared with PS80 PBCA NP/BDNF *p*DNA polyplex. Evidence for neural-lineage specification of mouse iPSCs was observed by an increased expression of nestin, neurofilament heavy polypeptide, and beta III tubulin, and the effects appeared superior when transfection was performed with the chitosan-coated formulation. This study illustrates the versatility of the PS80 PBCA NP and that surface decoration with chitosan enabled this delivery platform to be used for the transfection-guided differentiation of mouse iPSCs.

## 1. Introduction

Gene transfection represents a valuable tool for analyzing gene/protein regulation and function; it also provides a means to treat genetic disorders or acquired diseases by delivering therapeutic genetic material or regulatory elements into the patient’s cells [1,2,3]. Gene transfection can be mediated broadly by biological, chemical, or physical means [4]; viral vectors represent the most commonly employed biological method for stable and sustainable gene transfection, however, problems associated with insertional mutagenesis, immunogenicity, package size limitation, and hazards to laboratory personnel are well-known drawbacks of this method [5,6,7]. Alternatively, physical methods utilize various forms of mechanical forces to deliver genes into cells thus bypassing various extra- and intracellular barriers and circumvents endocytosis, yet these methods are still technically demanding, challenging to scale and limited by poor cell viability [8]; chemical methods make use of polymers or lipids as non-viral vectors to carry genetic materials in the form of polyplexes and lipoplexes, respectively, these methods may be less likely to cause insertional mutagenesis, immunogenicity, and injuries to cells, and is less restricted by package size, but may be associated with a lower transfection efficiency than viral vectors particularly for transfecting primary cells, progenitor cells, and stem cells [9]. Strategies that have been described to improve the gene transfection efficiency of chemical vectors can be broadly categorized into methods that increase its extracellular stability, cellular affinity and internalization, intracellular trafficking, and nuclear entry [10], many of which can be realized through a nanotechnological approach.

The nanotechnology-based colloidal drug delivery system is a versatile tool for gene delivery. The physicochemical characteristics, as well as decoration with specific functional ligands, can be specifically chosen for the purpose of gene transfection [11,12,13,14]. Nano-sized polymeric or lipid vectors have traditionally been designed to bear a positive charge, the carrying efficiency and the stability of the resultant polyplexes or lipoplexes are greatly increased because these cationic species easily form complexes with the anionic genetic cargo. The favorable interaction with the negatively charged proteoglycans on the cell membrane facilitates cell internalization by endocytosis, moreover, avoidance of intracellular degradation by endosomal escape and release of the genetic cargo may be enhanced by lipid neutralization with the endosome membrane anionic lipids, and the buffering capacity-related proton sponge effect in the case of cationic lipoplexes and polyplexes respectively. In our previous study, polybutylcyanoacrylate nanoparticles (PBCA NP) was used as a polymeric colloidal vector for brain-derived neurotrophic factor (BDNF) gene transfection and BDNF protein delivery; uptake of these agents by mouse induced pluripotent stem cells (iPSCs) resulted in neural-lineage-committed cells in as short as 7 days [15,16]. The biocompatible and biodegradable nature of PBCA NPs, as well as central nervous system targeting capabilities, conferred by polysorbate 80 (PS80) coating, makes it ideal as a delivery system for drugs, peptides, proteins, and genes into the central nervous system [17,18,19]. PBCA NPs can be synthesized under ambient conditions using scalable emulsion polymerization of n-butylcyanoacrylate monomers or nano-precipitation of pre-formed PBCA polymers [20], and the cargo can either be carried by surface adsorption on pre-formed nanoparticles or be encapsulated within the nanoparticle during its synthetic process. The size of the nanoparticle is adjustable depending on the synthetic method, with those produced by the emulsion polymerization generally ranging from 100 nm to 200 nm [21], and larger nanoparticles ranging from 200 nm to 300 nm produced by the nano-precipitation method [22]. While various functional moieties can be added through simple surface adsorption, surfactant- or polyethylene glycol-mediated adsorption, or direct conjugation [23], similarly, the usual negative surface charge of PBCA NPs can be modified by coating the nanoparticle with charged moieties such as diethylaminoethyl dextran, Eudragit® RL100 and chitosan [24,25,26].

Chitosan is a biocompatible and biodegradable linear polycationic polysaccharide composed of randomly distributed 1-4 linked 2-acetamido-2-deoxy-β-D-glucopyranose and 2-amino-2-deoxy-β-D-glucopyranose, it is the only naturally occurring cationic amino polysaccharide derived from the partial *N*-deacetylation of chitin [27]. Chitosan is soluble only in weak acids, but it is viscous in the aqueous form even at low concentrations. Moreover, its solubility in water is poor and correlates with the degree of deacetylation; the available molecular weights range from 3800 to 20,000 Da, and its amino group has a pKa value of 6.5 which undergoes protonation in acidic conditions, thus forming a polycation that readily complexes with a variety of anionic species such as synthetic polymers, lipids, proteins and DNA. Thus, it has been widely explored as a delivery platform in the biomedical field [28,29]. Nano-to-macro sized chitosan-based gene delivery particles can be fabricated either by polyelectrolyte complexation or ionic gelation; the former involves the self-assembly of particles from electrostatically coupled chitosan and nucleic acid, whereas in the latter anionic cross-linkers such as tripolyphosphate, thiamine pyrophosphate, sodium sulfate, and dextran sulfate are commonly employed to form a matrix entrapping the nucleic acid rather than relying on the electrostatic interaction between chitosan and nucleic acid, which confers greater stability for sustained release and enables the simultaneous entrapment of other therapeutic agents [30]. Both methods require fine-tuning, taking into account the reacting pH, the physicochemical characteristics, and concentration of chitosan; the ratio of nucleic acid and cross-likers, and physical agitation, however, generally yields particles of larger size and of greater size distribution.

In this study, we intend to optimize the transfection efficiency of mouse iPSCs using PS80 PBCA NP as a non-viral vector bearing the plasmid DNA (*p*DNA) encoding the brain-derived neurotrophic factor (BDNF) gene, modified with the hypoxia-responsive element and the cytomegalovirus promotor genes for neural-lineage commitment. Charge modification of the PS80 PBCA NP is accomplished by coating with chitosan. The influence of molecular weight, concentration of chitosan on the characteristics and cytotoxicity of PS80 PBCA NP is investigated, the *p*DNA loading efficiency and DNA protective effect of the polyplex is determined, and finally, the neural-lineage commitment of mouse iPSCs upon transfection with the chitosan-modified PS80 PBCA NP bearing the BDNF *p*DNA is assessed.

## 2. Results

The nanoparticle characteristics are summarized in Table 1. The average diameter (Dav) of the PS80 PBCA NP was the smallest which was measured at 120.5 ± 1.2 nm and was close to electrical neutrality with a zeta potential of −2.0 ± 0.1 mV. PS80 PBCA NP was also the most monodispersed preparation out of all with a polydispersity index (PDI) of 0.08 ± 0.03. Surface decoration of PS80 PBCA NP with 0.1% (*w*/*v*) of chitosan gave rise to an increase in Dav and zeta potential proportional to the molecular weight of the chitosan, which measured 238.0 ± 6.4 nm and 351.1 ± 1.1 nm, and 48.6 ± 0.6 mV and 50.0 ± 1.2 mV for low molecular weight chitosan- (LMWC) and high molecular weight chitosan- (HMWC) coated PS80 PBCA NPs, respectively. The PDI became unacceptably high when 0.1% (*w*/*v*) HMWC was used. Lowering the concentration of chitosan to 0.01% (*w*/*v*) effectively limited the size of the nanoparticles to below 150 nm; which measured 135.2 ± 0.4 nm and 149.3 ± 2.8 nm, and the zeta potential was reduced to 23.0 ± 1.0 mV and 33.9 ± 0.5 mV for LMWC and HMWC, respectively. The PDI was able to be kept at below 0.3 using 0.01% (*w*/*v*) of chitosan. Taking account of the Dav and PDI, the concentration of the chitosan for coating the PS80 PBCA NP was therefore chosen at 0.01% (*w*/*v*) for the synthesis of the BDNF *p*DNA polyplex.

The characteristics of the various BDNF *p*DNA-loaded PS80 PBCA NP polyplexes are summarized in Table 2. The zeta potential of the free BDNF *p*DNA, 0.01% (*w*/*v*) LMWC and HMWC molecules were −65.7 ± 2.0 mV, 48.3 ± 9.4 mV, and 53.2 ± 6.7 mV, respectively. Loading of the BDNF *p*DNA onto the nanoparticles resulted in electric charge neutralization and ultimately the formation of anionic polyplexes which measured −10.8 ± 2.2 mV, −31.4 ± 2.0 mV and, −34.8 ± 1.8 mV, respectively; polyplex formation was also associated with a mild elevation of particle Dav and PDI. The complexation by the polyelectrolyte method using 0.01% (*w*/*v*) LMWC and HMWC with BDNF *p*DNA gave rise to anionic polyplexes of approximately 200 nm and PDI of greater than 0.4.

**Figure 1:**
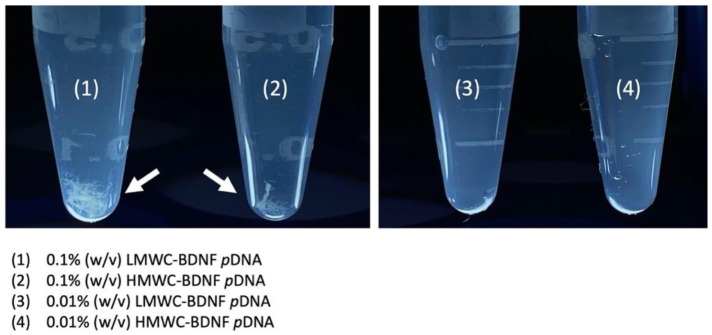
Gross examination of the products formed by the complexation of 0.1% (*w*/*v*) and 0.01% (*w*/*v*) of LMWC and HMWC and BDNF *p*DNA. Fibrillar precipitates (white arrows) were seen when 0.1% (*w*/*v*) of chitosan was used regardless of the molecular weight. No precipitates were observed with the 0.01% (*w*/*v*) chitosan.

The gross examination of the polyplexes formed by mixing 0.1% (*w*/*v*) and 0.01% (*w*/*v*) of LMWC and HMWC with BDNF *p*DNA is shown in Figure 1. Fibrillar precipitates were clearly seen when complexation was performed with the 0.1% (*w*/*v*) concentration regardless of the molecular weight of chitosan, by contrast, precipitation was not observed when the lower chitosan concentration was used.

**Figure 2:**
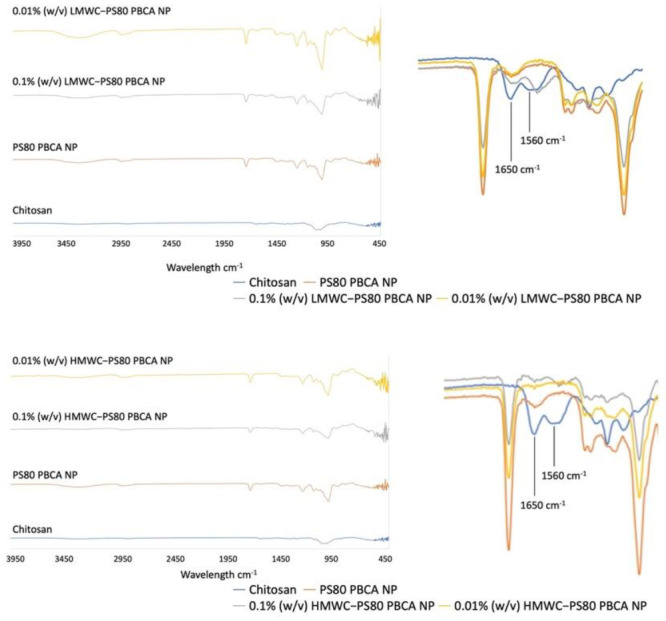
The attenuated total reflectance Fourier transform infrared spectroscopy of the PS80 PBCA NP surface-modified with 0.1% (*w*/*v*) and 0.01% (*w*/*v*) LMWC and HMWC revealed distinct absorption bands characteristic of chitosan (blue line) near the 1560 cm^−1^ and 1650 cm^−1^ regions (right panel of the figure), which were evident for nanoparticles coated with LMWC or HMWC of 0.1% (*w*/*v*, gray lines) but were undetectable for the 0.01% (*w*/*v*, yellow lines) concentration.

The attenuated total reflectance Fourier transform infrared spectroscopy (ATR-FTIR) of the chitosan-coated nanoparticles showed characteristic absorption bands for chitosan near 1560 cm^−1^ and 1650 cm^−1^ regions (Figure 2), which corresponded to the N-H bending of the primary amine and the stretching of C=O bonds of amide I in the presence of *N*-acetyl groups, respectively [31]. These bands were evident for both the LMWC- and HMWC-coated nanoparticles only when the concentration of 0.1% (*w*/*v*) was used, but was undetectable in the 0.01% (*w*/*v*) nanoparticle formulation.

**Figure 3:**
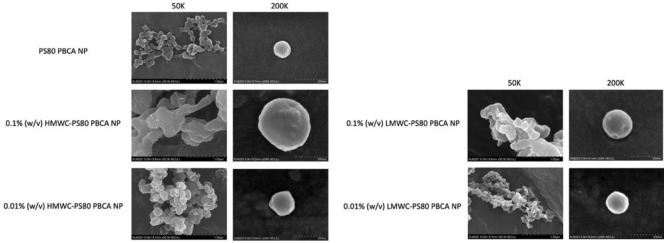
FE-SEM micrographs of PS80 PBCA NP, 0.1% (*w*/*v*) HMWC-PS80 PBCA NP, 0.1% (*w*/*v*) LMWC-PS80 PBCA NP, 0.01% (*w*/*v*) HMWC-PS80 PBCA NP, and 0.01% (*w*/*v*) LMWC-PS80 PBCA NP. 50,000x (solid samples) and 200,000x (aqueous samples) magnification. The shape of the nanoparticles were spherical in solution; the largest nanoparticle size was obtained when 0.1%, HMWC was used.

The field emission scanning electron microscope (FE-SEM) of the nanoparticles is shown in Figure 3. The nanoparticles appeared spherical at higher magnification using aqueous samples, the size of which was compatible with the results obtained by Zetasizer Nano ZS90. Coating the PS80 PBCA NP with 0.1% (*w*/*v*) chitosan led to an increase in particle size, which was more significant when HMWC was used. The size of the nanoparticles was kept below 150 nm when 0.01% (*w*/*v*) chitosan was used.

**Figure 4:**
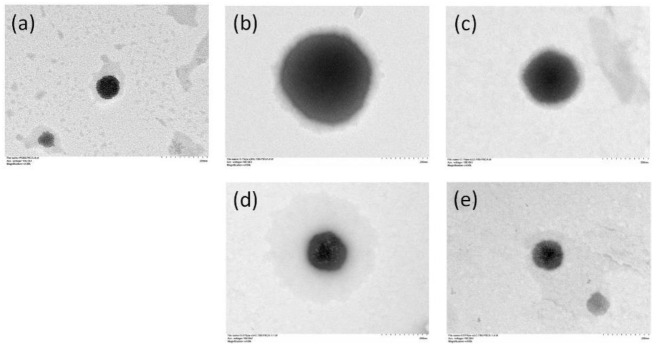
TEM micrographs of (**a**) PS80 PBCA NP, (**b**) 0.1% (*w*/*v*) HMWC-PS80 PBCA NP, (**c**) 0.1% (*w*/*v*) LMWC-PS80 PBCA NP, (**d**) 0.01% (*w*/*v*) HMWC-PS80 PBCA NP, and (**e**) 0.01% (*w*/*v*) LMWC-PS80 PBCA NP. 200,000×. Coating of the nanoparticles with chitosan resulted in a grayish halo and an ill-defined margin, the thickness of which appeared to increase with chitosan concentration and molecular weight.

The transmission electron microscope (TEM) images of the nanoparticles are shown in Figure 4. The bare PS80 PBCA NP appeared homogeneously electron-dense with a well-defined margin, however, an ill-defined grayish halo was observed when chitosan coating was performed. The thickness of the halo appeared to increase with the chitosan concentration and molecular weight.

**Figure 5:**
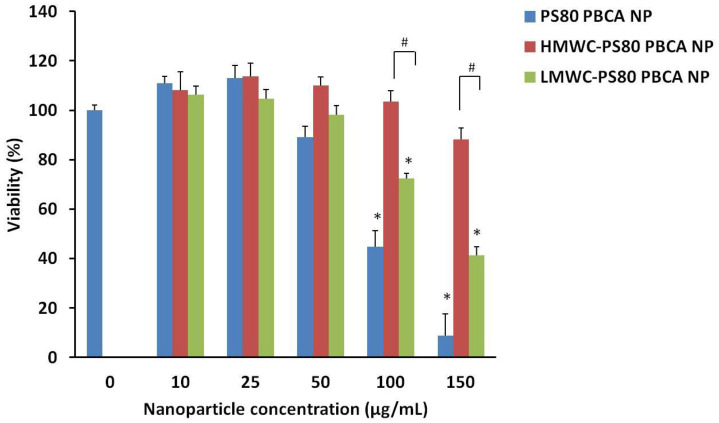
The cytotoxicity of mouse iPSCs to the nanoparticles at different concentrations was determined by the Cell Counting Kit-8. A cell viability of 90% or above was observed for 25 µg/mL of PS80 PBCA NP, 50 µg/mL of (0.01% *w*/*v*) LMWC-PS80 PBCA NP and 150 µg/mL of (0.01% *w*/*v*) HMWC-PS80 PBCA NP. N = 4; * *p* < 0.01, # *p* < 0.001.

The cytotoxicity of the nanoparticles on the mouse iPSCs was determined by the Cell Counting Kit-8 (CCK-8). A concentration-dependent cytotoxicity profile of the PS80 PBCA NP, LMWC-PS80 PBCA NP, and HMWC-PS80 PBCA NP is shown in Figure 5. The viability of mouse iPSCs was maintained at above 90% with the unmodified PS80 PBCA NP from concentrations of up to 25 µg/mL. By contrast, coating the nanoparticle with chitosan increased the cell tolerability to 50 µg/mL and 150 µg/mL for LMWC-PS80 PBCA NP and HMWC-PS80 PBCA NP, respectively. A significant reduction in cell viability was observed at concentrations above these limits. Complex formation with the BDNF *p*DNA in the following experiments was performed with the HMWC-PS80 PBCA NP, owing to its favorable cytotoxicity profile.

**Figure 6:**
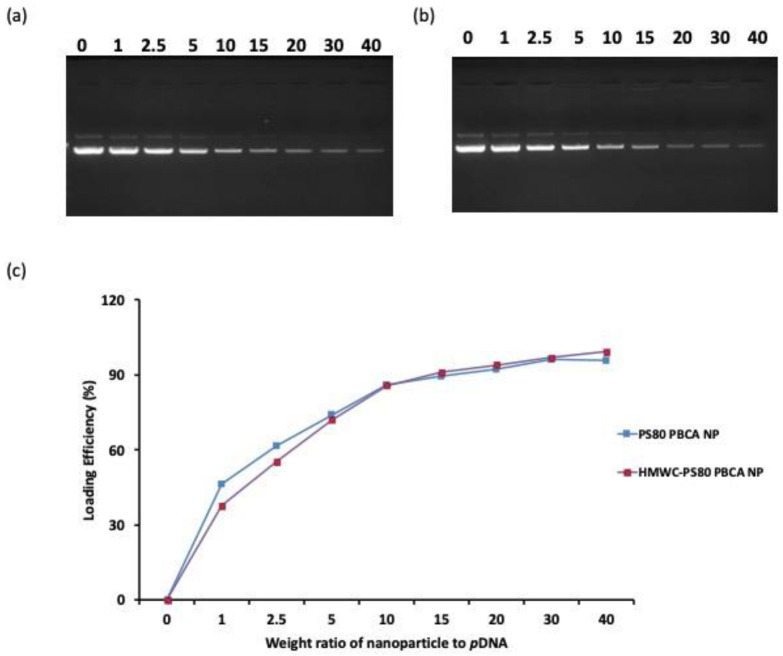
(**a**) Agarose gel electrophoresis of PS80 PBCA NP/BDNF *p*DNA and (**b**) HMWC-PS80 PBCA NP/BDNF *p*DNA polyplexes. A reduction in free BDNF *p*DNA was observed as the weight ratio of the nanoparticles to BDNF *p*DNA was increased. (**c**) The loading efficiency of BDNF *p*DNA on PS80 PBCA NP and HMWC-PS80 PBCA NP reached 90% at a weight ratio of 15 then plateaus.

Figure 6. shows the agarose gel electrophoresis of the supernatant after centrifugation of PS80 PBCA NP/BDNF *p*DNA and HMWC-PS80 PBCA NP/BDNF *p*DNA polyplexes to evaluate the quantity of unbound BDNF *p*DNA. The dose of the BDNF *p*DNA was chosen at 1 µg/mL, and the loading efficiency (LE) was subsequently determined for different weight ratios (from 1:1 to 40:1) of PS80 PBCA NP and HMWC-PS80 PBCA NP to BDNF *p*DNA. The LE for the two nanoparticles were approximately the same, which increased non-linearly with a rise in the weight ratio. For both nanoparticles, a weight ratio of 15:1 was associated with a high LE close to 90%, beyond which the LE appeared to plateau, thus, a weight ratio of 15 was selected for the polyplex synthesis.

**Figure 7:**
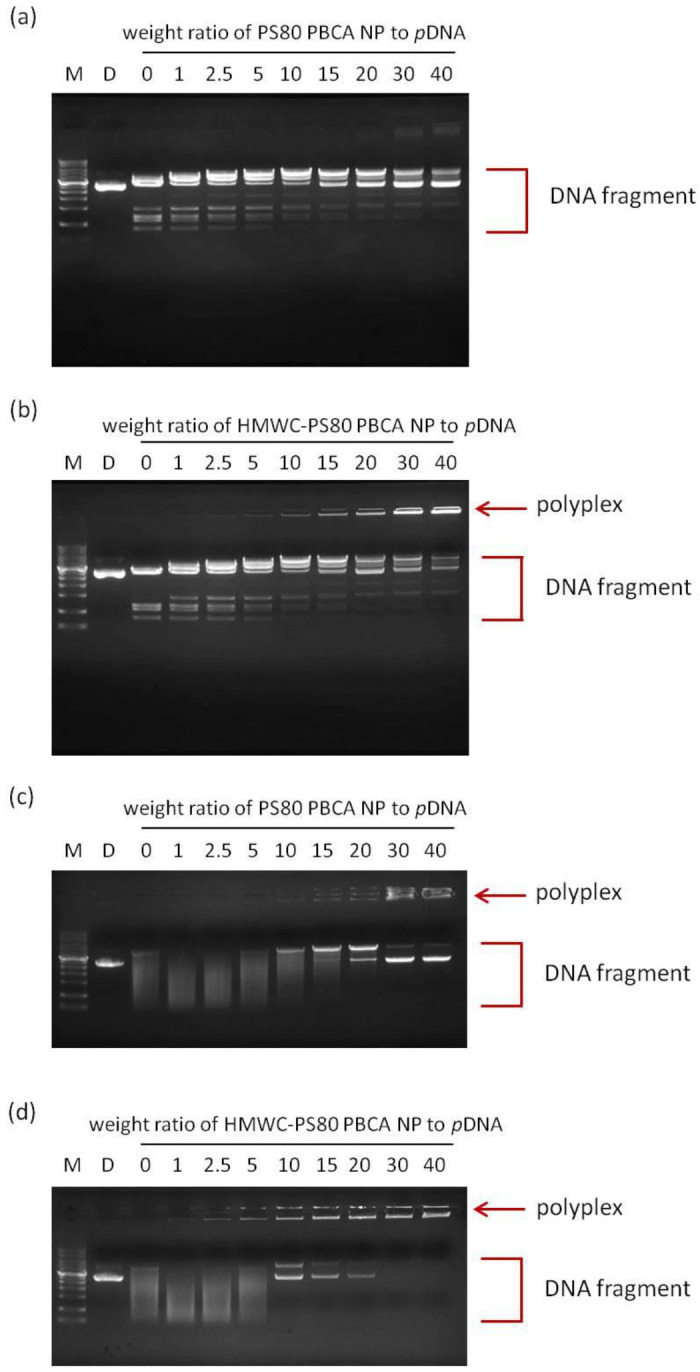
Agarose gel electrophoresis of PS80 PBCA NP/BDNF *p*DNA and HMWC-PS80 PBCA NP/ BDNF *p*DNA polyplexes after restriction with Bam HI/Hind III (**a**,**b**), and digestion with DNase I (**c**,**d**). M is the DNA maker and D is the BDNF *p*DNA. In the former group (**a**,**b**), free BDNF *p*DNA and its cleaved products were observed, and stable polyplexes were absent for PS80 PBCA NP/BDNF *p*DNA polyplex through the dose range tested, whereas stable polyplexes represented by the poorly electrophoretic bands were visible from a weight ratio of 10 and beyond when HMWC-PS80 PBCA NP/BDNF *p*DNA polyplex was used. In the latter group (**c**,**d**), stable polyplexes were visible in those made from PS80 PBCA NP only at a weight ratio of 10 and beyond, whereas these were found at a much lower weight ratio of 2.5 when HMWC-PS80 PBCA NP was used.

Figure 7a,b shows the agarose gel electrophoresis of PS80 PBCA NP/BDNF *p*DNA and HMWC-PS80 PBCA NP/BDNF *p*DNA polyplexes respectively after Bam HI/Hind III digestion. When the BDNF *p*DNA is mixed with PS80 PBCA NP or HMWC-PS80 PBCA NP, interaction with the nanoparticles will lead to the formation of polyplexes, the stability of which will influence the degree of digestion by the nucleases. The polyplex made with PS80 PBCA NP appeared less stable in the range of the weight ratio tested as free BDNF *p*DNA and its cleaved products were readily seen. On the other hand, the polyplex formed with HMWC-PS80 PBCA NP began to show a poorly electrophoretic band from a weight ratio of 10 and beyond, which indicated stabilization of the BDNF *p*DNA in the polyplex preventing nuclease digestion. Similarly, the agarose gel electrophoresis by the addition of DNase I into the PS80 PBCA NP/BDNF *p*DNA and HMWC-PS80 PBCA NP/BDNF *p*DNA polyplexes are shown in Figure 7c,d. Under these circumstances, the existence of stable polyplexes could only be seen in the former at a weight ratio of 10 and beyond, whereas, in the latter, stable polyplexes could be found at a weight ratio from as low as 2.5. Therefore, the polyplex with a weight ratio of 15:1 was chosen in the following experiments.

**Figure 8:**
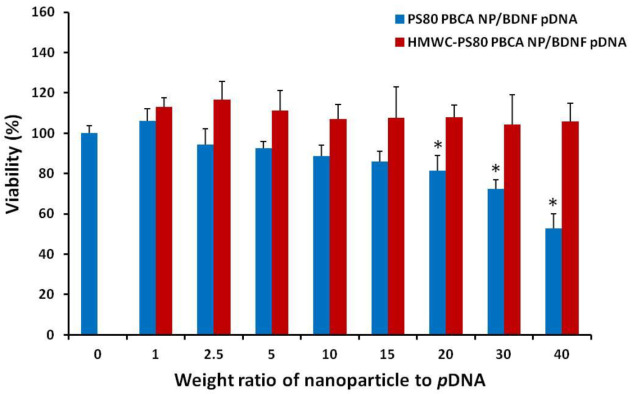
The cytotoxicity of mouse iPSCs to the polyplexes at different weight ratios. The HMWC-PS80 PBCA NP/BDNF *p*DNA polyplex remained non-toxic throughout the range of dose tested. By contrast, the viability of cells fell below 90% at a weight ratio of 10 for the PS80 PBCA NP/BDNF *p*DNA polyplex. *n* = 4; * *p* < 0.05.

The cytotoxicity of the polyplexes was tested on mouse iPSCs using the CCK-8 kit, and the result is displayed as a function of the weight ratio in Figure 8. The dose of the nanoparticles in the polyplexes corresponded to 2 µg/mL to 80 µg/mL, while the dose of *p*DNA was kept constant at 2 µg/mL. The HMWC-PS80 PBCA NP/BDNF *p*DNA polyplex remained non-toxic throughout the range of dose tested, however, significant cytotoxicity was found for PS80 PBCA NP/BDNF *p*DNA polyplex from a weight ratio of 10 and above.

**Figure 9:**
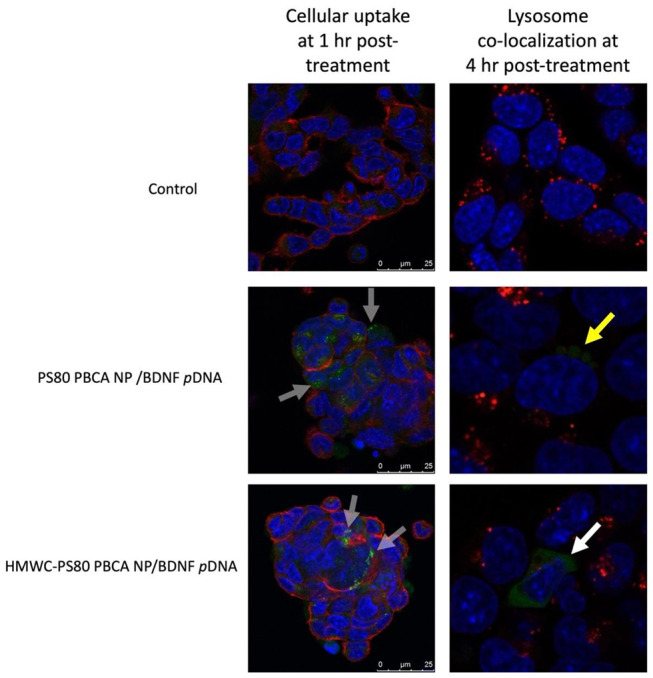
The cellular uptake (**left column**) and lysosome co-localization (**right column**) of FITC-dextran-tagged polyplexes were imaged by confocal microscopy. The cellular uptake study was performed at 1 h post-treatment with DAPI and WGA 594 for nuclear and cell membrane staining respectively, both PS80 PBCA NP/BDNF *p*DNA and HMWC-PS80 PBCA NP/BDNF *p*DNA polyplexes appeared as dense granules in the cytoplasm (gray arrows). The lysosome co-localization study was performed at 4 h post-treatment with Hoechst 33342 and LysoTraker™ Red DND-99 for nuclear and lysosome staining, respectively. PS80 PBCA NP/BDNF *p*DNA polyplex remained co-localized in the lysosome (yellow arrow), whereas HMWC-PS80 PBCA NP/BDNF *p*DNA appeared widely distributed in the cytoplasm (white arrow).

The cellular uptake of FITC-dextran-tagged polyplexes was imaged by confocal microscopy (Figure 9), first at 1 h post-treatment co-stained with DAPI and WGA 594 for nucleus and cell membrane, respectively, then at 4 h post-treatment co-stained with Hoechst 33342 and LysoTraker™ Red DND-99 for co-localization imaging. The imaging study revealed uptake of FITC-dextran-tagged PS80 PBCA NP/BDNF *p*DNA and HMWC-PS80 PBCA NP/BDNF *p*DNA polyplexes as small green-fluorescence granules in the cytoplasm after 1 h of treatment, however, co-localization imaging at 4 h post-treatment showed the co-location of PS80 PBCA NP/BDNF *p*DNA polyplex with the lysosome, whereas the fluorescence signals for HMWC-PS80 PBCA NP/BDNF *p*DNA appeared to be widely distributed in the cytoplasm without much lysosomal co-localization.

**Figure 10:**
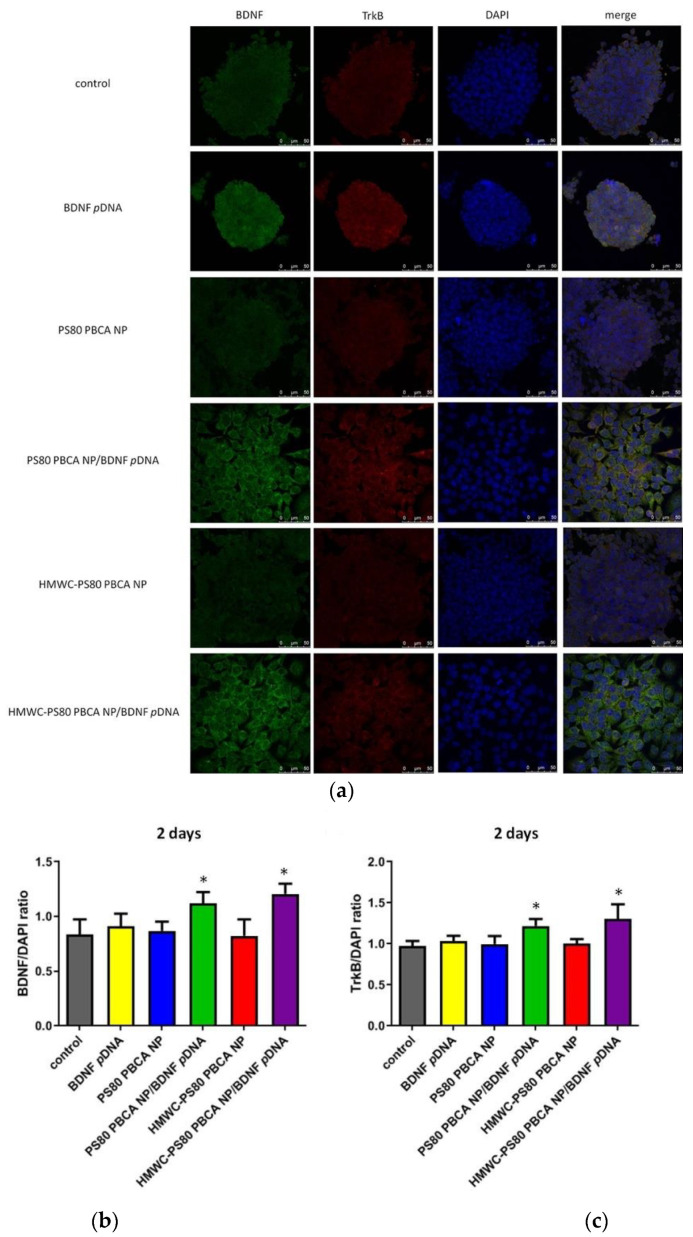

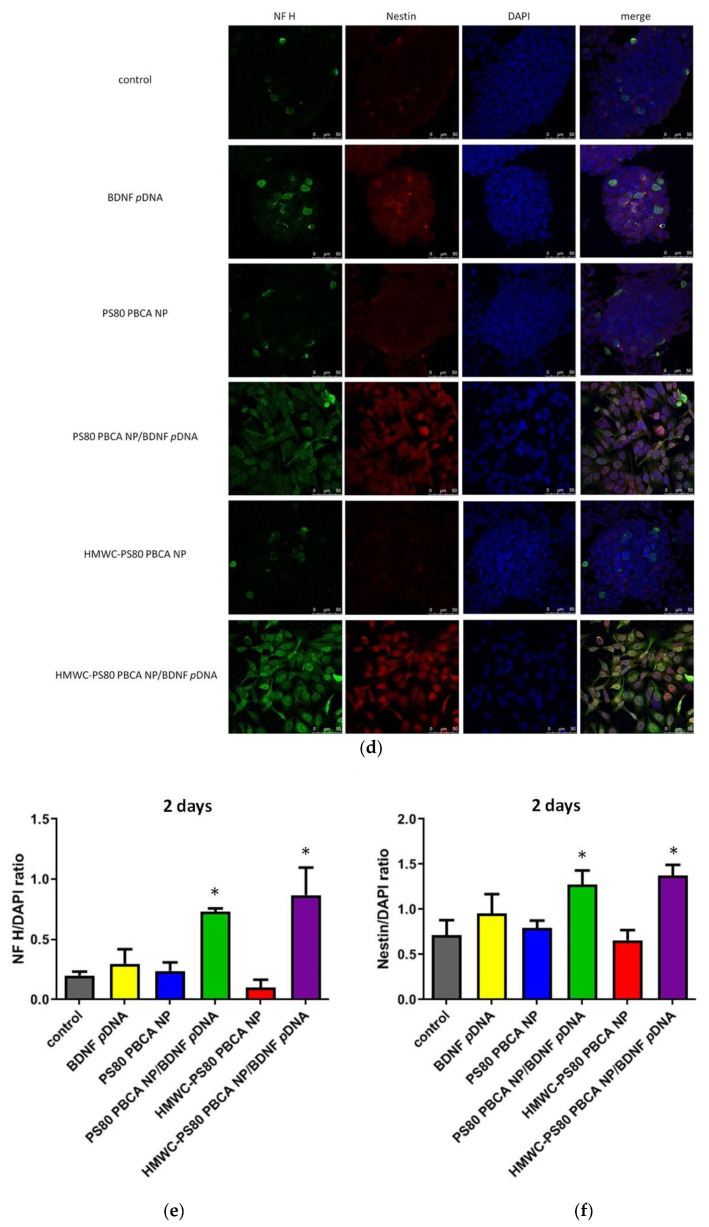

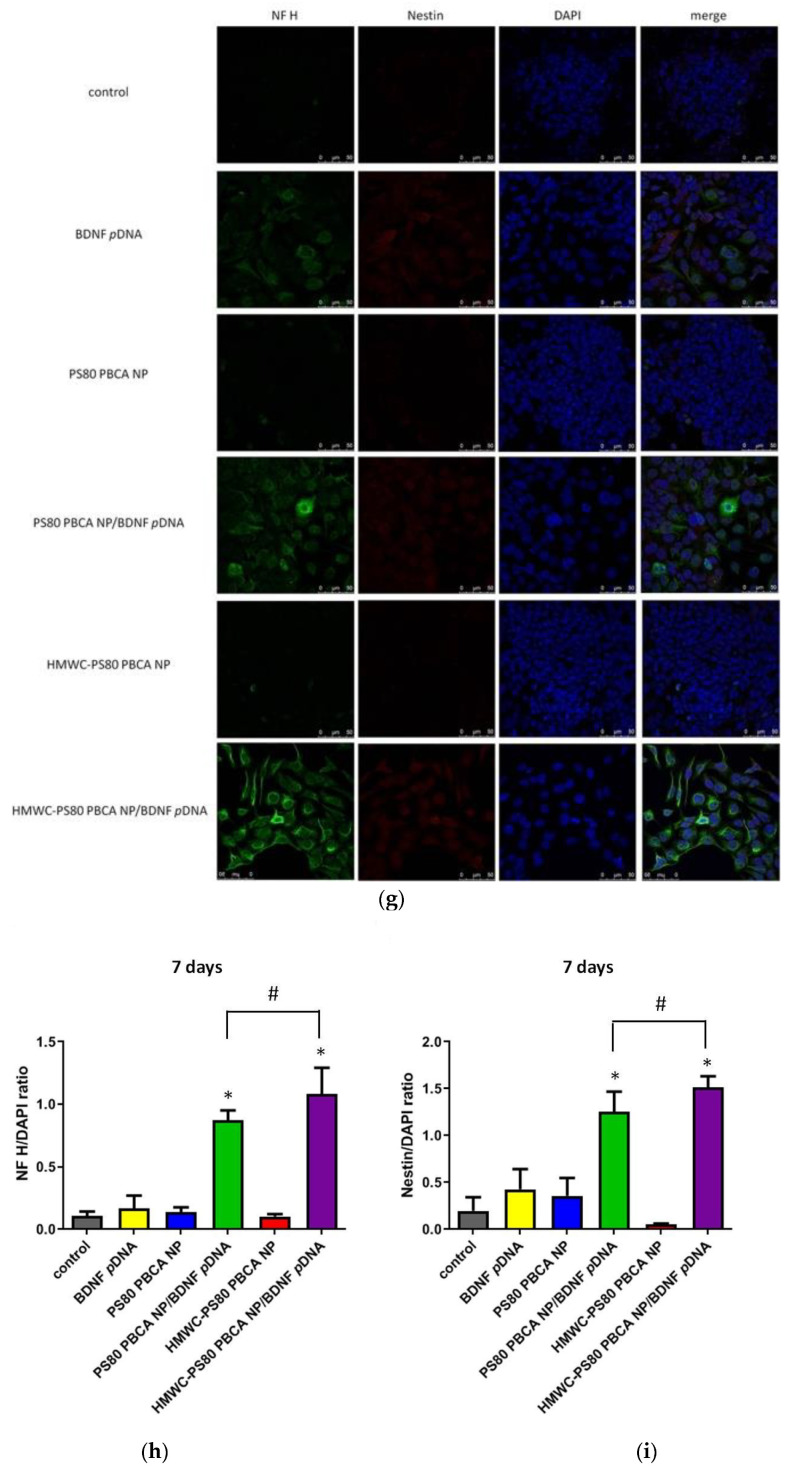
(**a**) Immunofluorescence staining of mouse iPSCs expressing BDNF and Tropomyosin-related kinase B (TrkB) at day-2 of transfection with prior exposure to hypoxia. Transfection was performed with BDNF *p*DNA (2 μg/mL), PS80 PBCA NP (30 μg/mL), PS80 PBCA NP/BDNF *p*DNA polyplex (30 μg/mL:2 μg/mL), HMWC-PS80 PBCA NP (30 μg/mL) or HMWC-PS80 PBCA NP/BDNF *p*DNA polyplex (30 μg/mL:2 μg/mL). (**b**) BDNF and (**c**) TrkB fluorescence intensities at day 2 post-transfection were significantly higher for the PS80 PBCA NP/BDNF *p*DNA and HMWC-PS80 PBCA NP/BDNF *p*DNA polyplexes to control but without obvious difference between the two. (**d**) Immunofluorescence staining for neurofilament heavy poly-peptide (NF H) and nestin following the same transfection conditions at day 2, and the respective fluorescence intensities for NF H (**e**) and nestin (**f**), the values were significantly higher for the two polyplexes without significant differences between them. (**g**) Immunofluorescence staining for NF H and nestin at day 7, and the respective fluorescence intensities for NF H (**h**) and nestin (**i**), both of which remained significantly higher for the HMWC-PS80 PBCA NP/BDNF *p*DNA. #, * *p* < 0.05.

The immunofluorescence staining for BDNF and its cognate receptor, tropomyosin-related kinase B (TrkB), neurofilament heavy polypeptide (NF H), and nestin at 2 days post-transfection with the polyplexes in mouse iPSCs pre-exposed to hypoxia are illustrated in Figure 10a,d. The immunofluorescence staining for NF H and nestin at 7 days post-transfection is shown in Figure 10g; the dose of the nanoparticles in the polyplexes was 30 µg/mL, while the dose of *p*DNA was 2 µg/mL (weight ratio = 15:1). The result showed absent to minimal expression of BDNF when the cells were exposed to free BDNF *p*DNA or the nanoparticles, and the cell colony remained densely packed with small indistinct cells. By contrast, the fluorescence staining for BDNF was clearly detectable when polyplexes were used (Figure 10b); the pattern of TrkB fluorescence coincided with those of BDNF (Figure 10c). Additionally, the cell colony appeared more loosely packed, and the cells were distinct, larger in size, and globoid in morphology. The immunofluorescence staining at 2 days post-transfection for nestin (a marker of neural stem/progenitor cells) and NF H (a marker of neuronal cells) were scant for the nanoparticles, but were occasionally seen for the free BDNF *p*DNA without an obvious change in the morphology of the cells or the cell colony; transfection with the polyplexes resulted in florid immunofluorescence of nestin and NF H, which was also associated with a change in the morphology of the cells and the cell colony (Figure 10d–f). The immunofluorescence signal for nestin was attenuated but remained strong for NF H at 7 days post-transfection but all in favor of the HMWC-PS80 PBCA NP/BDNF *p*DNA polyplex (Figure 10g–i).

**Figure 11:**
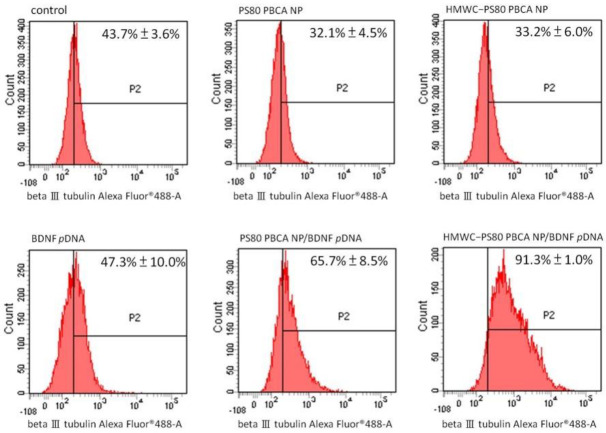
Flow cytometry analysis for the expression of neuron-specific beta III tubulin at day-7 post-transfection with the respective agents. Significant differences to the control in the proportion of beta III tubulin-positive cells were observed only for PS80 PBCA NP/BDNF *p*DNA and HMWC-PS80 PBCA NP/BDNF *p*DNA polyplexes. Furthermore, the latter formulation exhibited a significantly higher percentage than the former. *p* < 0.05.

Flow cytometry for the quantification of neuron-specific beta III tubulin-expressing cells is displayed in Figure 11. The result showed a significantly higher proportion of cells staining positively for beta III tubulin following transfection with the PS80 PBCA NP/BDNF *p*DNA and HMWC-PS80 PBCA NP/BDNF *p*DNA polyplexes than the control, which measured at 65.7 ± 8.5% and 91.3 ± 1.0%, respectively, and in favor with the polyplex containing chitosan.

## 3. Discussion

The modulation of cell function and behavior on a genetic level has crucial implications in molecular medicine and biotechnology; these include the treatment of certain inherited disorders, malignancies, and viral infections, the study of gene function, and genetic engineering of stem cells for regenerative therapy [4,32,33]. Unfortunately, the transfection of cells with free genetic material is inefficient owing to the electrostatic repulsive force that exists in between the negatively charged cell membrane and nucleic acids, and the presence of extracellular nucleases compromising its stability in the extracellular compartment [34]. Thus, gene delivery systems are a key requisite in order for gene transfection to occur. An ideal delivery system should be able to condense and protect the genetic material from degradation, minimally toxic to cells, traverse the cell membrane, escape from endo-lysosomal breakdown, and deliver the genetic material into the cell nucleus. Further considerations such as evasion of systemic clearance and bypassing the immune system are necessary for in vivo applications [35]; in the polymeric gene delivery system, this typically translates into the formation of stable nano-to-micro-sized polyplexes through the self-assembly of cationic polymers and DNA [36].

Chitosan has been used alone as a carrier for gene delivery, however, key challenges such as its poor water solubility, high viscosity, charge deduction at physiological pH leading to polyplex instability, and lack of targeting capability preclude its wide-spread use [30]. The molecular weight and the degree of deacetylation dictate the solubility, size and stability, cellular uptake, and cytotoxicity, which are all important design considerations for chitosan polyplex. In general, the solubility of chitosan increases with decreasing molecular weight and increasing degree of deacetylation [37], these parameters also impact the stability, cellular uptake, and cytotoxicity of the polyplexes through an alteration in surface charge which are all reduced upon a decrease in the molecular weight and the degree of deacetylation of chitosan. It has been reported that a molecular weight of at least 5 to 10 times larger than the genetic cargo is required in order for stable polyplexes to form, thus LMW to MMW and intermediate to high degree deacetylated chitosan is commonly chosen for the purpose of gene delivery [38,39,40,41]. In this study, 0.1% and 0.01% (*w*/*v*) HMWC were tested for its suitability as a carrier for BDNF *p*DNA; both were deemed unsuitable owing to the formation of gross fibrillar aggregates and moderately polydispersed particles of larger size, respectively. Thus, using PS80 PBCA NP as the core for surface modification by chitosan enabled better control of polyplex solubility, PDI, and size. The ATR-FITR spectroscopy of the chitosan-coated nanoparticles showed characteristic absorption bands for chitosan near 1560 cm^−1^ and 1650 cm^−1^ regions, even though these signals were only detectable with 0.1% (*w*/*v*) but not with 0.01% (*w*/*v*) chitosan, which might be related to the detection limit of this methodology, but the result from the dynamic light scattering, SEM and TEM, and DNA protection assay did indicate that the characteristics of the nanoparticles were modified when 0.01% (*w*/*v*) of chitosan was used.

We have demonstrated in our previous study the potential of PS80 PBCA NP as an anionic carrier for mouse iPSC transfection. We argued that the anionic polyplex might limit non-specific cellular uptake to avoid high levels of basal gene expression when the gene was coupled with a switchable mechanism such the hypoxia-responsive element, this could make the transfection process more site-specific and stimulus-driven, and the weak electrostatic interaction between the *p*DNA and the anionic PS80 PBCA NP might better facilitate desorption or unloading of the *p*DNA intracellularly for gene transcription [15]. In this study, PS80 PBCA NP was used to serve as a template for a polymeric gene delivery system by surface modification with chitosan; this in effect retains the positive features while overcoming the limitation in carrier synthesis from using chitosan alone. The increase in nanoparticle size and zeta potential was observed with the increase in molecular weight of chitosan, this is a trend that is in agreement with those reported by Sonvico et al. on the coating of poly-ε-caprolactone lipid-core nanocapsule [42]. On the other hand, higher chitosan concentration was unsuitable for coating due to an unacceptably high polydispersity index and large size of the nanoparticle, which was probably a result of flocculation from an excess of chitosan in the system [43]. In the case of mouse iPSCs, the cytotoxicity towards PS80 PBCA NP was attenuated by coating the nanoparticle with chitosan, especially with the HMW formulation. Therefore, taking all these factors together, 0.01% (*w*/*v*) HMWC was chosen as the coating agent for PS80 PBCA NP. The effect of chitosan-coating on the cytotoxicity of the PS80 PBCA NP is intriguing, although cationic nanoparticles are known to elicit membrane-permeabilizing effects that can irreversibly lead to cell damage and death [44], the exact structural conformation on the distribution of electric charge in the presence of PS80 PBCA NP, and together with the modification of electric charge by the interacting medium components as the protein corona evolves, may exert a stronger influence on its final cytotoxicity profile [45,46]. Additional factors other than electric charge can augment the cellular responses to nanoparticles, for example, the cytotoxicity of nanoparticles charge-modified by coating with chitosan has been shown to be dependent on the core material and stability of the coated nanoparticle, and the cell type and growth characteristics rather than the electric charge of chitosan alone [47,48].

The BDNF *p*DNA loading efficiency of bare PS80 PBCA NP appeared similar to HMWC-PS80 PBCA NP presumably from non-covalent interactions other than electrostatic forces, both agents were associated with a loading efficiency in excess of 90% at a nanoparticle to BDNF *p*DNA ratio of 15 and above. By contrast, the stability of the BDNF *p*DNA upon exposure to the nucleases such as BamHI, HindIII, and DNase I were different; the favorable electrostatic interaction enabled compaction of BDNF *p*DNA onto the cationic HMWC-PS80 PBCA NP and protects it against enzymatic digestion. The loading of BDNF *p*DNA onto the HMWC-PS80 PBCA NP resulted in the conversion of its positive surface charge into a negative charge—this may indicate the possibility of incomplete *p*DNA compaction or that additional *p*DNA may be carried through other interactions such as hydrogen bonding or hydrophobic forces [49]. The degree of DNA compaction is a function of the charge ratio, which is defined as the ratio of positively charged groups on the carrier to the negative charge on the DNA nucleotides, though important in the biologic medium, the reverse of DNA compaction deserves equal attention in order for the DNA to be released for transcription [50]. The variable protonation status of chitosan at different pH levels not only facilitates endo-lysosomal escape by the proton sponge effect, a process in which the amine groups are progressively protonated as the endo-lysosomal environment becomes increasingly acidified leading to an influx of water and chloride ions, and eventual osmotic swelling and rupture of endo-lysosome, but also a pH-responsive gene release system, in which the acid-stable polyplex undergoes DNA decompaction by deprotonation leading to a reduction in the adhesive forces and eventual release of the gene from its carrier as the pH rises close to neutral in the cytoplasm [51,52,53]. Although cationic polyplex facilitates cellular uptake, the membrane disruptive effect can lead to cytotoxicity and mitigate its utility, conversely anionic polyplexes are less cytotoxic but can still be internalized better than its counterpart under certain instances through adsorption onto cationic sites on the cell membrane. As a result, the reduced charge density on the plasma membrane allows the recruitment of other free particles to form clusters which are in turn internalized [54].

Stem cells represent an unlimited and renewable source of cells for cell therapy and tissue regeneration [55]. The basic steps of cell therapy consist of the derivation and expansion of stem cells, cell-lineage specification into progenitor cells or pre-differentiated cell products, and implantation of the cell product. Pluripotent stem cells derived from somatic cells or iPSCs not only bypass the ethical concerns of stem cell harvesting from the embryo but also avoids immune rejection by using an autologous cell source [56]. However, iatrogenic tumorigenesis from the persistence of iPSCs in the differentiated cell population remains a major problem for cell therapy using iPSCs [57,58]. The induction of iPSC differentiation entails complex differentiation steps with specific culture medium, growth factors, cytokines, and other supplements, transfection-guided differentiation may simplify and shorten this process [59,60]. In this study, the transfection of mouse iPCSc was in favor with the HMWC- PS80 PBCA NP/BDNF *p*DNA formulation possibly through the enhancement in the stability of the polyplex, endo-lysosomal escape, and intracellular release of the genetic cargo; the expression of BDNF and its cognate receptor TrkB as well as the neural stem/progenitor cell marker nestin, and neuronal marker NF H, by immunofluorescence staining, were observed on day-2 of gene transfection. This was coupled with morphologic changes in the cell colony; a decline in the expression of nestin was also found on day-7 post-transfection which is consistent with the temporal expression of nestin during neural development [61]. The elevated expression of another neuronal marker, beta III tubulin, by flow cytometry, also indicates the superiority of HMWC-PS80 PBCA NP/BDNF *p*DNA as the transfection agent.

## 4. Material and Methods

### 4.1. Materials

The low molecular weight chitosan (LMWC, 50–190 kDa, 75–85% deacetylation, Cat. No. 448869) and high molecular weight chitosan (HMWC, 310-375 kDa, >75 % deacetylation, Cat. No. 419419) were purchased from Sigma-Aldrich (St. Louis, MO, USA).

### 4.2. Construction of BDNF Plasmid DNA

The BDNF gene from codons 155 to 273 representing its functional domain was cloned and sequenced. The BDNF gene was amplified by the polymerase chain reaction (PCR) using the 5′-end PCR primers: 5′-GGATCCCACTCCGACCCCGCCCG-3′ and 5′-AAGCTTTCTTCCCCTTTTTAA TGGTCAGT-3′, coupled with the restriction sites for BamHI and Hind III respectively. The pSV-β-galactosidase control vector (Promega, Madison, WI, USA) was cut using the Hind III-NcoI restriction enzyme pair, and the CMV promotor isolated by Hind III digestion of the pGEM base (Promega) was inserted into the pSV-β-galactosidase control vector. The product was then digested with the BsaA I (New England BioLabs, Ipswich, MA, USA) and Ava I (New England BioLabs) restriction enzymes, and the amplified BDNF gene was inserted. The hypoxia-responsive element (P18 × 3) with the P18 sequence TGTCACGTCCTGCACGAC was inserted into the Sal I site of the pSV-β-galactosidase control vector. The constructed BDNF *p*DNA was subsequently transformed to the *Escherichia coli* JM 109 cells (ECOSTM 9-5; Yeastern Biotech, Taipei, Taiwan). The positive clones were selected by quick plasmid preparation and enzyme digestion, which were then verified by DNA sequencing.

### 4.3. Preparation of PS80-Coated PBCA NP and Modification with Chitosan, and Polyplex with the BDNF pDNA

PS80 PBCA NP was synthesized by the emulsion polymerization method as follows: 1% (*v/v*) butylcyanoacrylate monomers (BCA, Sicomet, Sichel Werk, Hanover, Germany) were added drop by drop into a 0.01 N HCl acidic polymerization solution containing 1% (*w*/*v*) dextran 70,000 (Sigma) and 0.1% (*v*/*v*) polysorbate 80 (PS80) at a pH of 2.0, stirred at 400 rpm and 25 °C for 3 h. The polymerization process was terminated by adding 0.1 N NaOH to the PS80 PBCA NP suspension. The suspension was centrifuged at 5250× *g* for 10 min and then filtered through a 0.45 μm filtration unit.

To further modify the PS80 PBCA NP with chitosan, 0.1% and 0.01% (*w*/*v*) solutions of LMWC and HMWC were prepared by dissolution in acetic acid, and then the PS80 PBCA NP was added to the 0.1% or 0.01% (*w*/*v*) chitosan solution, and stirred at 400 rpm for 30 min to allow adsorption of chitosan onto the surface of the PS80 PBCA NP. The samples were filtered through a 0.45 μm filtration unit and stored at 4 °C before use.

The loading of BDNF *p*DNA onto the uncoated or chitosan-coated PS80 PBCA NP to form polyplexes was performed by mixing the BDNF *p*DNA (from 1 mg/mL stock in DPBS buffer) with the respective nanoparticles (from 2 mg/mL stock in DPBS buffer) at various weight ratios for 1 h at room temperature. For comparison, polyplex formation was also assessed by mixing equal concentrations of BDNF *p*DNA in 0.1% and 0.01% (*w*/*v*) of LMWC and HMWC.

### 4.4. Characterization of the Nanoparticles and Polyplexes

#### 4.4.1. Particle Size and Zeta Potential

The cumulant Z-average diameter (Dav), zeta potential (ζ), and polydispersity index (PDI) of the nanoparticles and polyplexes were determined by a Zetasizer Nano ZS90 (Malvern, Worcestershire, UK) with a photo correlation spectroscopy and a laser Doppler velocimeter at 25 °C.

#### 4.4.2. Attenuated Total Reflectance Fourier Transform Infrared Spectroscopy Analysis of the Chitosan-Coated PS80 PBCA NPs

The ATR-FTIR spectra of LMWC, HMWC, PS80 PBCA NP, and chitosan-coated PS80 PBCA NPs using different concentrations of LMWC and HMWC were obtained using the PerkinElmer Spectrum 1000 spectrometer (Thermo Fisher Scientific, Waltham, MA, USA) equipped with the ATR accessory. The dried samples were analyzed sequentially with the cleaning of the crystal mount in between tests. The spectra were recorded with a resolution of 4 cm^−1^ over the range of 4000–450 cm^−1^ at a constant temperature of 25 °C.

#### 4.4.3. Morphology of the Nanoparticles and Polyplexes

The aqueous sample consisting of nanoparticles or polyplexes was placed on a copper grid in a specimen holder which was vacuum-dried and sputtered with platinum at 2 kV for 90 s. Then, a field emission scanning electron microscope (FE-SEM, SU-8220, Hitachi, Tokyo, Japan) was used to determine the surface features of the particle at 200,000× magnification; solid sample on a carbon adhesive tape was used for 50,000× magnification. Transmission electron microscopy (TEM, H-7500, Hitachi Koki, Tokyo, Japan) was performed following negative staining with 2% (*w*/*v*) phosphotungstic acid (PTA, Sigma) to visualize the morphology and the internal structure of the particle.

### 4.5. Loading Efficiency of BDNF pDNA on the Nanoparticles

The loading efficiency (LE) of *p*DNA on the nanoparticles was evaluated by determining the fraction of unbound to nanoparticle-bound *p*DNA in the nanoparticle solution at different weight ratios of nanoparticles to *p*DNA after 1 h of incubation at room temperature. The samples were centrifuged at 16,100× *g* for 30 min and an ND-1000 spectrophotometer (NanoDrop; Thermo Fisher Scientific) was used to quantify the amount of free *p*DNA in the supernatant by 260 nm light absorbance. The LE was calculated as: LE (%) = [(total weight of *p*DNA − weight of free *p*DNA)/(total weight of *p*DNA)] × 100%.

Free *p*DNA in the supernatant was analyzed by electrophoresis on a 0.8% agarose gel (Sigma) with 1× TAE buffer (Amresco, Solon, OH, USA) at 50 V for 30 min. *p*DNA was visualized by staining the gels with ethidium bromide (0.5 mg/mL, Sigma) and illuminated using a Syngene U: Genius Gel Image System (Cambridge, UK).

### 4.6. BDNF pDNA Protection Assay

The polyplexes prepared from using different weight ratios (1:1, 2.5:1, 5:1, 10:1, 15:1, 20:1, 30:1, and 40:1) of PS80 PBCA NP or chitosan-coated PS80 PBCA NP to *p*DNA were tested with the *p*DNA protection assay, which involved the digestion of the carrier-bound *p*DNA by the restriction enzyme (Bam HI/Hind III) or nuclease (DNase I). Briefly, the polyplex (containing 1 μg *p*DNA) was incubated with 1 μL of Bam HI (1 U/μL, Thermo Fisher Scientific) and 1 μL of Hind III (1 U/μL, NEB) in 50 μL of reaction buffer (50 mM Tris-HCl, 10 mM MgCl_2_, 100 mM NaCl, 0.1 mg/mL BSA) at 37°C for 30 min, and then the *p*DNA fragments was analyzed by gel electrophoresis (0.8% agarose). Similarly, the polyplex (containing 1 μg *p*DNA) was incubated with 1 μL of RNase-free DNase I solution (0.05 U/μL, Thermo Fisher Scientific) in 50 μL of reaction buffer (10 mM Tris-HCl, 2.5 mM MgCl_2_, 100 μM CaCl_2_) at 37 °C for 10 min, and then the integrity of the *p*DNA was analyzed by gel electrophoresis (0.8% agarose).

### 4.7. Cell Cultivation

The mouse iPSCs (SC201A-iPSCs, System Biosciences, Palo Alto, CA, USA) were maintained in ESGRO complete plus clone grade medium (Millipore, Billerica, MA, USA) containing selective GSK3β inhibitor supplement (Millipore), and cultivated in a humidified CO_2_ incubator (Thermo Fisher Scientific) at 37 °C.

### 4.8. Cellular Uptake and Lysosome Co-Localization of the Polyplexes

The cellular uptake of the polyplexes was performed by first seeding the mouse iPSCs on 24-well microplates at a density of 1 × 10^4^ cells/cm^2^ and cultured overnight then treated with FITC-dextran-labelled (Sigma) PS80 PBCA NP/BDNF *p*DNA or HMWC-PS80 PBCA NP/BDNF *p*DNA at a weight ratio of 15:1 for 1 h, washed with 1× DPBS and fixed in 4% paraformaldehyde; 5 µg/mL of wheat germ agglutinin (WGA) 594 (Thermo Fisher Scientific) and 1 µg/mL DAPI (Sigma) were added and incubated for 10 min at room temperature and washed with 1× DPBS for confocal microscopy (Leica TCS SP5 II, Wetzlar, Germany) with the excitation and emission wavelengths set at 490 nm and 520 nm for FITC-dextran, 590 nm and 617 nm for WGA 594, and 341 nm and 452 nm for DAPI.

The lysosome co-localization of the polyplexes was performed by seeding 1 × 10^5^ cells on 35 × 12 mm glass dishes and incubated overnight then treated with the respective polyplexes for 4 h, the medium was changed with those containing 5 µg/mL of Hoechst 33342 (Thermo Fisher Scientific) and 37.5 nM of LysoTracker™ Red DND-99 (Thermo Fisher Scientific), and incubated for 30 min at 37 °C and 5% CO_2_. The medium was changed before confocal microscopy, the excitation and emission wavelengths were set at 490 nm and 520 nm for FITC-dextran, 577 nm and 590 nm for LysoTracker™ Red DND-99, and 350 nm and 461 nm for Hoechst 33342.

### 4.9. Cytotoxicity of the Nanoparticles and Polyplexes

The cytotoxicity of mouse iPSCs to the nanoparticles and polyplexes was assessed with the Cell Counting Kit-8 (CCK-8; TargetMOL, MA, USA). The cells were seeded on 96-well polystyrene microplates (Corning Inc., Corning, NY, USA) at a density of 1 × 10^4^ cells/well and incubated for 16 h. The cells were then treated for 4 h with various concentrations of the nanoparticles or polyplexes in 100 μL of culture medium and incubated for 24 h. The assay was performed by adding 10 μL CCK-8 solution to each well and incubated for 3 h, then the optical absorbance at 450 nm of the reduced product (formazan) was obtained with the EnSpire Multimode Plate Reader (PerkinElmer). The quantitative data are presented as optical density (OD) and are taken as a direct measure of the number of viable cells. The cytotoxicity of the nanoparticles and the polyplexes was defined as the relative viability of the treated cells to the untreated control.

### 4.10. Protein Expression after Gene Transfection by Immunofluorescence Staining

Gene transfection with the polyplexes on mouse iPSCs was performed as follows: mouse iPSCs were seeded 24 h before transfection into 24-well plates at a density of 1 × 10^5^ cells/cm^2^. Then, the cells were incubated under hypoxia (5% CO_2_, 1% O_2,_ 94% N_2_, and 95% relative humidity) at 37 °C for 4 h before gene transfection. The polyplexes were made first by adding 30 μg/mL of PS80 PBCA NP or chitosan-modified PS80 PBCA NP to 2 μg of BDNF *p*DNA for 1 h and ESGRO complete plus clone grade medium was then added to make up a 1 mL solution; this volume was given to a single well of cells. Gene transfection was carried out at 37 °C and 5% CO_2_ for 4 h. After that, the medium was replaced and the cells were incubated under normoxia for 2 or 7 days. The experiment was repeated with 30 μg/mL PS80 PBCA NP and 2 μg of BDNF *p*DNA for comparison.

The cells were fixed with 10% formalin solution for 10 min, and treated with 0.1% Triton-X-100 in DPBS for 10 min to improve the membrane permeability. The sample was incubated with CAS-Block (Life technologies, Frederick, MD, USA) for 10 min to reduce non-specific background staining. Primary antibodies against BDNF (1:1000, Abcam), TrkB (1:200, Abcam), neurofilament heavy (NF H; 1:200, Abcam), and nestin (1:200, Abcam) were added and placed at 4 °C overnight. The sample was washed with PBS and secondary antibodies conjugated to Alexa Fluor^®^ 488 Alexa (1:1000, Abcam) or Alexa Fluor^®^ 594 Alexa (1:1000, Abcam) were applied for 1 h at room temperature. The nuclei of the cells were counterstained with DAPI. Immunofluorescence was observed by a confocal laser scanning microscope. The fluorescence intensity of BDNF, TrkB, NF H, and nestin normalized to those of DAPI was semi-quantified by imageJ analysis.

### 4.11. Flow Cytometry for Neuron-Specific Beta III Tubilin

The detection of neuron-specific beta III tubulin-positive cells by flow cytometry was done by first seeding 1 × 10^4^/cm^2^ of mouse iPSCs on 10 cm polystyrene dishes and cultured overnight, then hypoxic stimulus was given by incubation under 5% CO_2_, 1% O_2,_ 94% N_2_, and 95% relative humidity at 37 °C for 4 h, and treatment for 4 h with PS80 PBCA NP, HMWC-PS80 PBCA NP, BDNF pDNA, PS80 PBCA NP/BDNF pDNA, HMWC-PS80 PBCA NP/BDNF pDNA were performed followed by a further incubation period under normoxia for 7 days. The cells were fixed with 4% paraformaldehyde and treated with 0.1% Triton-X-100 in DPBS for 10 min. Staining was carried out first with anti-beta III tubulin antibody (1:100, Abcam, Cambridge, UK) under room temperature for 30 min, and with secondary antibodies conjugated to Alexa Fluor^®^ 488 Alexa (1:1000, Abcam). The fluorescence-labeled cells were placed in 200 µL of DPBS with 1% (*w*/*v*) formaldehyde at 4 °C and passed through a polystyrene round-bottom tube with a cell-strainer cap (Corning Inc.). Flow cytometry analysis was carried out by a BD FACSCanto™ II flow cytometer (BD Biosciences, Franklin Lakes, NJ, USA) and analyzed with Cell Quest software (BD Biosciences).

### 4.12. Statistics

Data were presented as the mean with standard deviation. All statistical analyses were performed by using the Stata version 11.0 statistical software (StataCorp LLC, College Station, TX, USA). The data were analyzed with the two-way analysis of variance (ANOVA) and Tukey’s HSD test. A *p*-value of 0.05 or less was taken as a significant statistical difference.

## 5. Conclusions

The non-viral nano-scaled biocompatible polymeric gene delivery system using PS80 PBCA NP as the backbone for surface modification with chitosan was constructed. The study showed that surface modification with chitosan retained the positive characteristics of chitosan as a delivery vector, while issues associated with poor solubility and cytotoxicity were eliminated. The delivery system enabled greater polyplex stability against DNA digestion, endo-lysosomal escape, and enhanced gene expression in comparison to the polyplex formed without the use of chitosan, this allowed a shortened preparatory period for neural lineage specification of mouse iPSCs.

## Figures and Tables

**Table 1:** The average diameter (Dav), zeta potential, and polydispersity index (PDI) of the nanoparticles.

Nanocarrier	Dav (nm)	Zeta Potential (mV)	PDI
PS80 PBCA NP	120.5 ± 1.2	-2.0 ± 0.1	0.08 ± 0.03
0.1% (*w*/*v*) HMWC-PS80 PBCA NP	353.1 ± 1.1	50.0 ± 1.2	0.62 ± 0.02
0.1% (*w*/*v*) LMWC-PS80 PBCA NP	238.0 ± 6.4	48.6 ± 0.6	0.34 ± 0.01
0.01% (*w*/*v*) HMWC-PS80 PBCA NP	149.3 ± 2.8	33.9 ± 0.5	0.29 ± 0.03
0.01% (*w*/*v*) LMWC-PS80 PBCA NP	135.2 ± 0.4	23.0 ± 1.0	0.19 ± 0.01
N = 6; *p* < 0.001			

**Table 2:** The average diameter (Dav), zeta potential, and polydispersity index (PDI) of the polyplexes prepared at a weight ratio of nanoparticle to *p*DNA of 15:1.

Nanocarrier	Dav (nm)	Zeta Potential (mV)	PDI
PS80 PBCA NP/BDNF *p*DNA	129.2 ± 0.3	−10.8 ± 2.2	0.17 ± 0.01
0.01% (*w*/*v*) HMWC-PS80 PBCA NP/BDNF *p*DNA	163.8 ± 1.8	−34.8 ± 1.8	0.33 ± 0.01
0.01% (*w*/*v*) LMWC-PS80 PBCA NP/BDNF *p*DNA	148.5 ± 1.8	−31.4 ± 2.0	0.33 ± 0.01
0.01% (*w*/*v*) HMWC-BDNF *p*DNA	204.4 ± 17.0	−45.6 ± 1.6	0.51 ± 0.09
0.01% (*w*/*v*) LMWC-BDNF *p*DNA	205.5 ± 5.5	−40.9 ± 1.3	0.43 ± 0.08
BDNF *p*DNA	-	−65.7 ± 2.0	-
0.01% (*w*/*v*) HMWC	-	53.2 ± 6.7	-
0.01% (*w*/*v*) LMWC	-	48.3 ± 9.4	-
N = 6; *p* < 0.001			

## Data Availability

Not applicable.
